# Mutations in the Mitochondrial *ND1* Gene Are Associated with Postoperative Prognosis of Localized Renal Cell Carcinoma

**DOI:** 10.3390/ijms17122049

**Published:** 2016-12-07

**Authors:** Hakushi Kim, Tomoyoshi Komiyama, Chie Inomoto, Hiroshi Kamiguchi, Hiroshi Kajiwara, Hiroyuki Kobayashi, Naoya Nakamura, Toshiro Terachi

**Affiliations:** 1 Department of Urology, Tokai University School of Medicine, Kanagawa, Isehara 259-1193, Japan; terachit@is.icc.u-tokai.ac.jp; 2 Department of Clinical Pharmacology, Tokai University School of Medicine, Kanagawa, Isehara 259-1193, Japan; komiyama@tokai-u.jp (T.K.); hkobayas@is.icc.u-tokai.ac.jp (H.Ko.); 3 Department of Pathology, Tokai University School of Medicine, Kanagawa, Isehara 259-1193, Japan; cisophia.ci@gmail.com (C.I.); h-kaji@is.icc.u-tokai.ac.jp (H.Ka.); naoya@is.icc.u-tokai.ac.jp (N.N.); 4 Support Center for Medical Research and Education, Tokai University, Kanagawa, Isehara 259-1193, Japan; kamiguchi@is.icc.u-tokai.ac.jp

**Keywords:** mitochondrial DNA, *ND1*, renal cell carcinoma, prognostic factor, polymorphism, heteroplasmy, nucleotide differentiation index

## Abstract

We analyzed mutations in the mitochondrial *ND1* gene to determine their association with clinicopathological parameters and postoperative recurrence of renal cell carcinoma (RCC) in Japanese patients. Among 62 RCC cases for which tumor pathology was confirmed by histopathology, *ND1* sequencing revealed the presence of 30 mutation sites in 19 cases. Most mutations were heteroplasmic, with 16 of 19 cases harboring one or more heteroplasmic sites. Additionally, 12 sites had amino acid mutations, which were frequent in 10 of the cases. The 5-year recurrence-free survival (RFS) rate was significantly worse in patients with tumors >40 mm in diameter (*p* = 0.0091), pathological T (pT) stage ≥3 (*p* = 0.0122), Fuhrman nuclear atypia grade ≥III (*p* = 0.0070), and *ND1* mutations (*p* = 0.0006). Multivariate analysis using these factors revealed that mutations in *ND1* were significantly associated with the 5-year RFS rate (*p* = 0.0044). These results suggest a strong correlation between the presence of *ND1* mutations in cancer tissue and postoperative recurrence of localized RCC in Japanese patients.

## 1. Introduction

Kidney cancer is globally the 9th and 14th most common malignant tumor among males and females, respectively [1]. In developed countries, renal cell carcinoma (RCC) constitutes 85%–90% of all cancers of the kidney [2,3]. There is a 1.5:1 predominance of new RCC cases in males over females, and the peak age of onset is between 60 and 70 years [4]. The advent and widespread use of abdominal imaging techniques, such as abdominal ultrasonography and computed tomography, has led to the increased frequency of the diagnosis of small asymptomatic RCCs, which account for about half of all newly diagnosed cases [5]. Despite the increase in the number of new cases, the mortality rate due to RCC has, in fact, decreased [6,7]. However, even in patients with localized RCC that has been resected with curative surgery, such as total or partial nephrectomy, the relapse rate is about 20%–40%, with progression to metastatic RCC (mRCC) [8].

These observations suggest that even small RCCs can exhibit aggressive metastatic ability to distant organs [9]. In addition, given the lack of an effective adjuvant therapy to reduce the risk of recurrence after curative surgery, early detection and intervention in patients with recurrent RCC is critical to improving prognosis in mRCC. However, there are no established prognostic models to accurately predict the metastatic potential of RCCs. Recent studies suggested that several prognostic models that were developed based on the TNM system may not effectively predict the postoperative prognosis of distinct RCC types, and the development of improved models with increased accuracy for recurrence patterns is sorely needed [10,11,12]. Additionally, diagnostic and prognostic molecular markers for RCC have not yet been fully incorporated into clinical practice [13,14].

Human mitochondrial DNA (mtDNA) is a closed, circular molecule comprising 16,569 nucleotide pairs and encoding 13 essential genes with roles in oxidative phosphorylation as well as the structural rRNA and tRNAs necessary for the expression of these genes [15]. mtDNA is encoded separately from nuclear DNA and is in close proximity to reactive oxygen species (ROS) due to functional necessity. For this reason, and as histone proteins that protect nuclear DNA are missing in mitochondria, mtDNA accumulates mutations at a higher rate than nuclear DNA, which subsequently affect the oxidative phosphorylation capacity of mitochondria [16].

Recent studies proposed that mutations in mtDNA might be utilized as molecular markers for early detection, assessment of the degree of malignancy, and prediction of recurrence and prognosis after radical surgery in several cancer types. [17,18]. mtDNA mutations that resulted in ROS accumulation were shown to be correlated with the acquisition of metastatic potential and poor prognosis [19]. For example, Bai et al. demonstrated the association of a nine-base pair deletion at nucleotide position 8272 (region V) of the mtDNA with postoperative survival rate in RCC [20]. In addition, mutations in NADH dehydrogenase subunit 1 (*ND1*), a subunit of oxidative phosphorylation complex, were detected in breast and nasopharyngeal carcinomas [21,22]. Furthermore, Yusnita et al. [23] identified 11 missense mutations in *ND1* from colorectal tumor tissue specimens. Studies also showed that mutations in *ND1* may lead to changes in the secondary structure of the protein and subsequent inactivation of enzymes [23]. The studies described above indicate that there was a correlation between various types of cancer and the *ND1* mutations, which may lead to subsequent abrogation of its enzymatic activity [21,22,23]. These studies provide strong evidence for the utility of mtDNA mutations as potential prognostic factors for recurrence and progression in various types of cancer.

In this study, we aimed to determine the correlation between mtDNA mutations and postoperative recurrence in RCC. We performed comparative sequence analysis of mtDNA extracted from formalin-fixed paraffin-embedded (FFPE) RCC and the paired non-cancerous tissue specimens to evaluate postoperative gene markers as prognostic factors.

## 2. Results

### 2.1. Clinical Characteristics of Study Subjects

Characteristics of patients and tumor specimens are summarized in Table 1. Median age was 62 (39–80) years, and male-to-female ratio was 3.4:1. Median follow-up period was 66.5 (29–76) months. From 62 subjects in this study that were followed up with for a minimum of two years, 11 had recurrent RCC by June 2016. Among pathological characteristics, median tumor diameter in the surgical specimen was 32 (12–105) mm. Histologically, 55 patients had clear cell RCC (ccRCC), whereas tumors were non-clear cell RCC (nccRCC) in the remaining seven cases. By histopathological diagnosis, tumors were locally confined (stage pT1–2) in 57 patients, whereas tumors invaded the vascular or surrounding tissue (pathological T (pT) stage 3–4) in five patients. By Fuhrman grading of nuclear atypia, 54 and eight cases were grade I/II and III/IV, respectively. Microvessel invasion was observed in 12 patients.

### 2.2. Nucleotide Differentiation Index Analysis (N_ST_)

In this study comprising 62 Japanese patients with RCC, we investigated the *ND1* gene using the nucleotide differentiation index (*N*_ST_) [24]. As controls, *ND1* sequences of 62 healthy Japanese subjects were obtained from GenBank. We determined that the *N*_ST_ was 0.005, indicating that RCC was significantly associated with mutations in the mitochondrial *ND1* gene.

### 2.3. Analysis of ND1 Mutations

**Figure 1:**
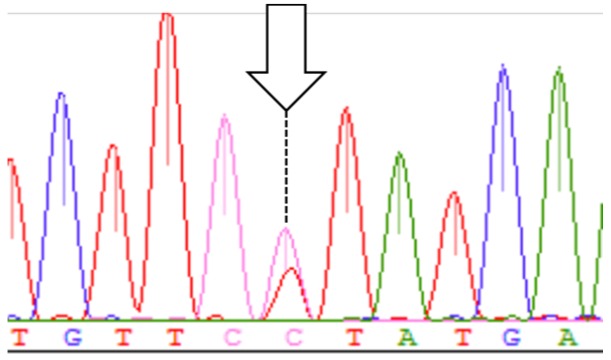
The signal of the heteroplasmic mutation site.

We analyzed the *ND1* sequences (956 base pairs) using the FFPE tissue specimens of 62 patients with RCC. We found somatic mutations, including heteroplasmy, at 30 sites in 19 cases (30.6%; 19/62 patients) (Figure 1). Only one site (3970) was confirmed to have both homoplasmic and heteroplasmic mutations. In addition, C3328Y, C4197Y, and C4141Y with heteroplasmic mutations were novel mutations that were not previously reported in Japanese patients. Moreover, mutations leading to amino acid changes were observed at 12 sites in 10 cases (52.6%, 10/19 patients). The *ND1* region of patient hk383 harbored a mutation, insertion of a C at position 3572, which caused a frameshift. Heteroplasmic mutations were observed at 24 sites in 16 cases (84.2%, 16/19 patients), and 84% of the cases with mutations harbored heteroplasmic mutations (Table 2). Since mtDNA is generally homoplasmic, heteroplasmic mutations observed in this cohort were considered to have occurred as part of changes in conditions related to RCC.

### 2.4. Analysis of Recurrence Risk Factors and Cancer-Specific and Overall Survival

**Figure 2:**
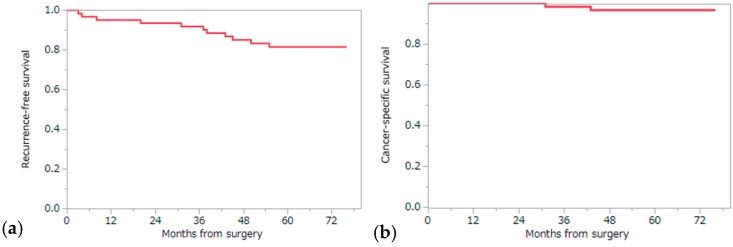
Kaplan–Meier curve for the 5-year RFS (**a**) and CSS (**b**) of a total of 62 patients included in this study.

**Figure 3:**
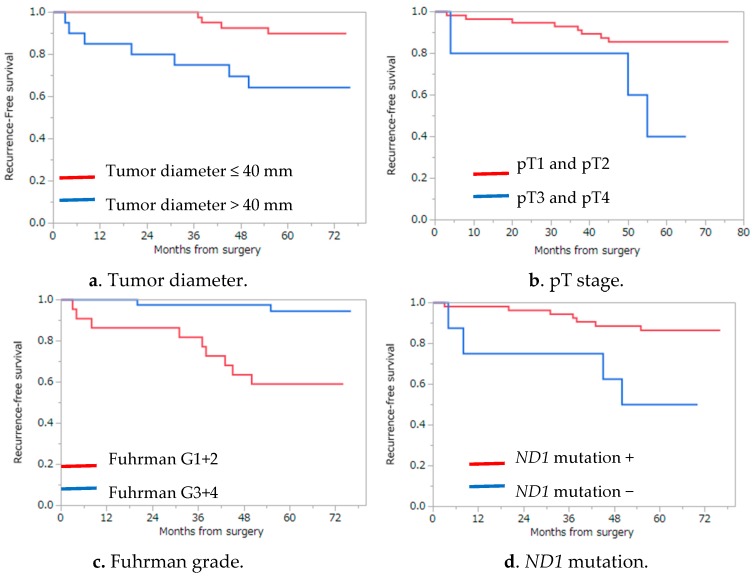
Results of Kaplan–Meier analysis for RFS, which showed statistically significant differences by the log-rank test. (**a**) tumor diameter (*p* = 0.0091); (**b**) pT stage (*p* = 0.0122); (**c**) Fuhrman grade (*p* = 0.0070); and (**d**) *ND1* mutation (*p* = 0.0006) were candidates as postoperative prognostic factors for localized renal cell carcinoma.

The 5-year recurrence-free survival (RFS), cancer specific survival (CSS), and overall survival (OS) were 81.6%, 96.7%, and 93.4% by the Kaplan–Meier analysis, respectively (Figure 2). Among the ten factors that were assessed by the log-rank test, four factors (maximum tumor diameter, pT stage, Fuhrman grade, and *ND1* mutation) were significantly associated with recurrence, suggesting that these factors might impact RFS: tumor diameter (5-year RFS, 89.9% and 64.3% for ≤40 and >40 mm, respectively; *p* = 0.0091), pT stage (5-year RFS, 85.6% and 40.0% for ≤pT2 and ≥pT3, respectively; *p* = 0.012), Fuhrman grade (5-year RFS, 86.6% and 50.0% for I/II and III/IV, respectively; *p* = 0.0070), and somatic mutation in *ND1* (5-year RFS, 59.1% and 94.6% for the presence and absence of somatic mutation/s in *ND1*, respectively; *p* = 0.0006) (Table 3) (Figure 3). Multivariate analysis using the Cox proportional hazards model using these four factors showed that only the presence of somatic mutations in the *ND1* gene was a significant factor associated with RFS (*p* = 0.0044) (Table 4). We also examined the association of these ten factors with CSS using the log-rank test. As a result, three factors (maximum tumor diameter, pT stage, and *ND1* mutation) were found to be significantly associated with CSS: tumor diameter (5-year CSS, 100% and 90% for ≤40 mm and >40 mm, respectively; *p* = 0.0415), pT stage (5-year CSS, 98.2% and 80.0% for ≤pT2 and ≥pT3, respectively; *p* = 0.0330), and somatic mutation in *ND1* (5-year CSS, 89.5% and 100% for the presence and absence of somatic mutation/s in *ND1*, respectively; *p* = 0.0341) (Table 5). Multivariate analysis using the Cox proportional hazards model for these three factors showed that there was no factor significantly associated with CSS; however, somatic mutation in *ND1* showed a tendency toward deterioration of CSS in the presence of *ND1* mutation (*p* = 0.0827) (Table 6). These findings suggest that mutations in *ND1* might also be considered as a prognostic factor for recurrence and a possible predictor of CSS. Among the ccRCC subgroup (55 cases), we examined the association of RFS with the same nine factors using the log-rank test and Cox proportional hazards model. As a result of the log-rank test, three factors (maximum tumor diameter (*p* = 0.0161), Fuhrman grade (*p* = 0.0051), and *ND1* mutation (*p* = 0.0042)) were candidates of becoming independent predictors of RFS. Finally, *ND1* mutation was the only statistically significant predictive factor of RFS in the ccRCC sub group according to the Cox proportional hazards model (*p* = 0.0208) (Table 7).

## 3. Discussion

In this study, we conducted a comparative sequence analysis of mtDNA extracted from FFPE RCC specimens and the paired non-cancerous tissue specimens to determine postoperative genetic markers as potential prognostic factors for the recurrence of RCC. As a result, 30 mutations were found in the *ND1* sequence of 19 patients. These mutations were not found in healthy Japanese subjects based on the GenBank database and were confirmed to be somatic by comparing with the paired non-cancerous tissue specimens. Majority of the mutations were heteroplasmic; 16 of 19 cases had one or more heteroplasmic sites. Heteroplasmy suggests the presence of a pathogenic mutation as a cause or result of cancer and was previously shown to have a strong association with mutations [25,26,27]. In six cases, there were three new mutation sites (C3328Y, C4141Y, and C4197Y) that were not previously reported and were not found in the database. Of these new mutation sites, C3328Y and C4141Y were missense mutations, whereas L8F and R279W were amino acid changes resulting from the mutations. With respect to amino acid changes, we found mutations that led to changes in amino acids at 12 sites in 10 cases (52.6%; 10/19 patients). Mutations that lead to amino acid changes may alter protein structures and subsequently affect their function and activity.

We identified an insertion of C at position 3572 (C3572ins) in patient hk383, which caused a frameshift and an amino acid change. With this insertion, the protein structure of *ND1* and complex I function may be significantly altered [28]. This specific mutation in mitochondrial *ND1* has been reported in oncocytic thyroid carcinoma [29], oncocytic pituitary adenoma [30], renal oncocytoma, and chromophobe RCC (eosinophilic variant) [28], suggesting that C3572ins mutation in *ND1* is associated with oncocytic tumors or variants. Oncocytic is derived from the granular eosinophilic appearance of cytoplasm due to the abnormal accumulation of mitochondria [28]. The patient with the C3572ins mutation (case hk383) also had a granular eosinophilic cytoplasm and was diagnosed with an eosinophilic variant of ccRCC. While a correlation between oncocytosis and tumorigenesis has been suggested [31], the relationship between C3572ins and tumor progression is not clear. In this study, our patient with the C3572ins mutation had metastatic recurrence in the lymph nodes and the adrenal glands eight months after surgery. However, further studies are needed to assess the utility of this mutation as a predictor for disease recurrence.

Our log-rank test revealed that tumor diameter >40 mm, pT stage≥ 3, Fuhrman grade ≥III, and *ND1* mutation were associated with worse RFS rates in patients who underwent surgery for localized RCC. Multivariate analysis using these factors revealed that mutations in *ND1* were significantly associated with RFS rates. Although larger tumor size, pT stage, and Fuhrman grade were previously reported as poor prognostic factors [32,33], this is the first study to show that mutations in *ND1* were predictors of recurrence in localized RCC.

The mechanism of carcinogenesis in RCC has not yet been elucidated; however, genetic factors were shown to be crucial to its development. For example, mutations of the von Hippel-Lindau (*VHL*) gene on the short arm of chromosome 3 and another locus on the long arm of chromosome 5 were shown to be associated with the carcinogenesis of RCC and are considered as candidate tumor suppressor genes [34,35]. However, factors that determine the speed and progression of RCC or its metastasis are not yet known. In this study, we focused on mitochondria, in particular, since its relationship with oxidative stress and consequent mtDNA mutations might affect the prognosis of patients with RCC. Mitochondria has a leading role in critical cellular functions such as glucose and lipid metabolism and apoptosis [36]. Furthermore, mitochondria has endogenous angiogenic factors, such as VEGF and TGF-β1, and VHL protein for protein degradation by ubiquitination [37,38]. The loss of *VHL* protein observed in most ccRCC cases suggest that it has contributions to the downregulation of biosynthesis of the oxidative phosphorylation complex. Solid tumors endure a severely hypoxic environment, and adaptation to such a hypoxic environment is a critical step in tumor progression. Increases in markers of oxidative stress, such as ROS and nitric oxide, were found in some patients with RCC [39]. In addition, patients with increased parameters of oxidative stress and decreased antioxidant levels were associated with higher grade, stage and metastatic disease [39].

This is the first study that demonstrates the relationship between mitochondrial *ND1* mutations and postoperative recurrence of RCC in a Japanese patient cohort. The Japanese are a single ethnic group and thus tend to show similar responses to treatments such as that observed with interferon therapy for RCC [40] and hepatocellular carcinoma [41]. Additionally, Japanese patients were recently shown to exhibit side effects that were distinct from those observed in Western populations in response to targeted therapy for RCC [42]. Furthermore, mtDNA sequences are stored in databases based on race and continent, and comparison of mtDNA sequences across different ethnic groups and races based on the findings of this study will be useful in determining the prognosis of RCC in Japanese patients in future studies. Finally, our findings showing that over 80% of the mutations found were heteroplasmic provide very strong evidence that mutations in *ND1* can be used as indicators of cancer prognosis in the future [43]. Studies with increased numbers of patients are necessary to confirm and improve the reliability of our findings in the present study.

## 4. Materials and Methods

### 4.1. Patients and Tumor Specimens

In this study, FFPE tissue specimens with accompanying clinical data were collected from a total of 62 patients who consecutively underwent radical surgery for localized RCC (clinical T1-4N0M0) at Tokai University School of Medicine Hospital between January and December 2010 and were retrospectively followed for a minimum of two years. This study was approved by the clinical research review committee of the Tokai University School of Medicine.

### 4.2. Assessment of Formalin-Fixed Paraffin-Embedded Tissue Specimens

Two pathologists microscopically confirmed the location of cancerous tissue and the paired non-cancerous tissue within the FFPE specimens, and tissue punch samples that were obtained with an 18 G needle were in separate 1.5-mL test tubes.

### 4.3. Amplification of mtDNA with Polymerase Chain Reaction

FFPE tissue punch samples were washed three times with 500 μL lemosol and three times with 99.5% ethanol to remove lemosol. Tissues were next incubated with 200 μg proteinase K in HMW Buffer (10 mM Tris-Cl pH 8.0, 150 mM NaCl, 10 mM EDTA (ethylenediaminetetraacetic acid), 0.1% SDS (sodium dodecyl sulfate)) at 60 °C for overnight, followed by extraction with phenol-chloroform (Phenol/Chloroform/Isoamyl alcohol, 25:24:1), each twice, at 11,000 rpm at room temperature for 10 min. DNA was precipitated by the addition of 0.1 vol 3 M Na-AcOH and 2.5 vol ice-cold ethanol. After centrifugation at 15,000 rpm at 4 °C for 20 min, DNA pellets were rinsed with 70% cold ethanol, dried, and dissolved in TE buffer (10 mM Tris-HCl, pH 8.0 and 1 mM EDTA). Total mtDNA was amplified by polymerase chain reaction (PCR) with the following primer sets that were designed to cover the entire 957-bp mtDNA *ND1* region:

F3274 (5′-ACAGTCAGAGGTTCAATTCCTCTTCT-3′),
R3590 (5′-ATAGGAGGCCTAGGTTGAGGTTGACCA-3′),
F3590 (5′-TGGTCAACCTCAACCTAGGCCTCCTAT-3′),
R3725 (5′-GATGGCTAGGGTGACTTCATATGAGA-3′),
F3731 (5′-ATGAAGTCACCCTAGCCATCATTCTACTA-3′),
R4021 (5′-TCATATGTTGTTCCTAGGAAGATTGTAGT-3′),
ND1-F (5′-TCCGAACTAGTCTCAGGCTTCA-3′),
ND1-R (5′-CACGGAGAATTTTGGATTCTCAG-3′).

PCR was performed by Ex Taq^®^ (Takara Bio, Tokyo, Japan) under the following conditions: melting at 95 °C for 10 s, annealing at 55 °C for 20 s, and extension at 72 °C for 30 s, for a total of 35 cycles. PCR products were treated by EXOSAP-IT^®^ (Affimetrix, Santa Clara, CA, USA) and directly sequenced using the Big Dye^®^ Terminator v3.1 Reaction Kit (Applied Biosystems, Torrance, CA, USA) mix and ABI 3500xL DNA sequencer (Life Technologies, Carlsbad, CA, USA). We also confirmed the sequences in the *ND1* region three times to confirm the mutations. The *ND1* DNA sequence data were assembled using the ATCG software (Genetyx, Tokyo, Japan). Accession numbers for the nucleotide sequences of *ND1* obtained from 62 patients included in the present study are LC178840–LC178901.

### 4.4. Nucleotide Differentiation Index Analysis

For *N*_ST_ analysis, nucleotide sequences of the *ND1* gene of 62 subjects in this study were aligned by ClustalW using the Molecular Evolutionary Genetics Analysis (MEGA, version 6, Tempe, AZ, USA, Pennsylvania, USA, Tokyo, Japan) software [24,44]. Common sites in all sequences with gaps were excluded from analysis. mtDNA sequences were compared with the reference mtDNA from 62 healthy long-living Japanese individuals from the Genbank database (Table S1). Mutation sites were used in homology searches across a range of databases (NCBI-dbSNP, MitoMap, Ensemble, JSNP), and NC_012920.1 (GI: 251831106) was chosen as the reference sequence for *ND1*. Next, 27 segregation sites from *ND1* gene sequences were selected. *N_STij_* for the *i*-site and the *j*^th^ subpopulation was defined for the *N*_ST_ analysis (Table S2). The *N*_ST_ formula was as follows [24]:

$$N_{STij}=\frac{H_{Ti}-H_{Sij}}{H_{Ti}}$$

### 4.5. Detection of Somatic Mutations in ND1 Gene

The mtDNA sequences of 62 healthy long-living Japanese individuals were randomly extracted from the Genbank database (Table S1) and were compared with the mtDNA sequences of RCC tissues. Subsequently, mutation sites in the mtDNA sequences of RCC tissues were extracted and determined. By extracting the sequence data of the non-cancerous renal tissue of the same patient, the mutations in the RCC tissue were confirmed to be unique and somatic. In addition, after examining the frequency of these mutations using the MtMap and Genbank databases, they were regarded as mutations in carcinoma tissues.

### 4.6. Statistical Analysis

JMP^®^ version 12.0.1 (SAS Institute, Cary, NC, USA) was used for all statistical analyses. RFS, CSS, and OS were calculated by the Kaplan–Meier method. Nine clinical and pathological factors were selected to determine their association with RFS and CSS, categorical variables were compared using the chi-square test, and the log-rank test was performed to examine the differences in their effect on RFS and CSS. The nine factors considered were as follows: age (≤60 years vs. >60 years), sex (male vs. female), nephrectomy (full vs. partial), tumor diameter (≤40 mm vs. >40 mm), histology type (ccRCC vs. nccRCC), pT stage (≤pT2 vs. ≥pT3), Fuhrman grade (I/II vs. III/IV), microvessel invasion (absence or presence), and *ND1* mutations (presence or absence). The median age was used for separating the younger patients from the older ones. As the prognosis of ccRCC, which comprises 70% of all RCC cases, is unfavorable compared with other RCC types, other histological RCC types were included in the nccRCC group for comparison with ccRCC (Table 1). We also analyzed the predictive factors of RFS among the ccRCC subgroup. Multivariate analysis using the Cox proportional hazards model was performed for candidate risk factors, which were statistically significant by the log-rank test. Furthermore, the independence of factors that influenced RFS and CSS was examined. *p* values less than 0.05 were considered as statistically significant.

## 5. Conclusions

This is the first study to demonstrate a relationship between mutations in *ND1* and postoperative recurrence of RCC in Japanese patients. Using microscopic evaluation of FFPE specimens, pathologists confirmed the status of tumors from which DNA was extracted. The *N*_ST_ value of 0.005 indicated that RCC was significantly associated with mutations in the mitochondrial *ND1* gene. Moreover, we identified 30 mutation sites in 19 patients. Most of the mutations were heteroplasmy, and 16 out of the 19 patients harbored one or more heteroplasmic sites. Additionally, 12 sites had mutations that led to amino acid change mutations and were frequent in 10 out of the 19 patients. Furthermore, the RFS was significantly worse in patients with larger tumors with a diameter of over >40 mm, those with ≥pT stage 3, those with Fuhrman grade ≥III, and those with *ND1* mutations in RCC specimens. Moreover, multivariate analysis revealed that the presence of *ND1* mutations was significantly associated with worse RFS.

## Figures and Tables

**Table 1:** Patient and tumor characteristics of 62 cases with localized renal cell carcinoma.

Age (years), Median (range)	62 (39–80)
Sex (male/female)	48/14
Nephrectomy (total/partial)	42/20
Observation (months), median (range)	66.5 (29–76)
Tumor diameter (mm), median, range	32 (12–105)
Histology type	
clear	55
papillary	2
chromophobe	3
multilocular	1
acquired cystic disease (ACD)-associated	1
Pathological T stage	
T1a	40
T1b	14
T2a	3
T2b	0
T3a	1
T3b	3
T4	1
Fuhrman	
G1	1
G2	53
G3	6
G4	2
Microvessel invasion	
V0	50
V1	12

**Table 2:** Nucleotide (single nucleotide mutation or heteroplasmic change) and amino acid changes in the *ND1* gene.

Patient	Base Change	AA Change	Heteroplasmy	Histology Type	Outcome
hk347	C4197Y *		+	Clear cell	No recurrence
	T4248Y		+		
hk355	C3497T	A64V		Clear cell	No recurrence
hk357	C4197Y *		+	Clear cell	No recurrence
hk362	C3970Y		+	Clear cell	No recurrence
hk363	T4248Y		+	Clear cell	No recurrence
hk364	C3497T	A64V		Clear cell	No recurrence
	G3635A	S110N			
hk368	C4197Y *		+	Clear cell	No recurrence
	T4248Y		+		
hk372	A4200W		+	Clear cell	Recurrence
	T4216Y	Y304H	+		
hk382	A4200W		+	Clear cell	No recurrence
	T4216Y	Y304H	+		
hk383	C3572ins	L89P (Frameshift)		Clear cell	Recurrence
hk385	G3496T	A64S		Clear cell	Recurrence/Cancer Death
	C4141Y *	R279W	+		
	T4248Y		+		
hk387	C4197Y *		+	Clear cell	Recurrence
	T4248Y		+		
hk392	G4048R	D248N	+	ACD-associated RCC	Recurrence
	C4071Y		+		
hk393	T3368Y	M21T	+	Clear cell	No recurrence
hk394	A4200W		+	Clear cell	Recurrence
	T4216Y	Y304H	+		
hk399	C3970Y		+	Clear cell	Recurrence
hk401	T4117Y		+	Clear cell	No recurrence (Figure 1)
hk403	C3328Y *	L8F	+	Clear cell	Recurrence/Cancer Death
	C3970T				
hk405	G4113R		+	Clear cell	No recurrence

* Novel mutations not previously reported in the Japanese population.

**Table 3:** Univariate analysis of factors predicting recurrence after surgery.

Variables	No. of Cases	5-Year RFS Rate (%)	*p* Value
Age (years)			0.1736
≤60	28	88.7	
>60	34	75.7	
Sex			0.2690
Female	14	91.7	
Male	48	78.7	
Tumor localization			0.7795
Left	24	83.1	
Right	38	80.4	
Nephrectomy			0.2951
Total	42	78.1	
Partial	20	89.2	
Tumor diameter (mm)			0.0091 **
≤40	42	89.9	
>40	20	64.3	
Histological type			0.9142
clear	56	81.6	
non-clear	6	80.0	
pT stage			0.0122 **
≤pT2	57	85.6	
≥pT3	5	40.0	
Fuhrman grade			0.0070 **
I/II	54	86.6	
III/IV	8	50.0	
Vessel invasion			0.1389
Presence	50	66.7	
Absence	12	85.5	
*ND1* somatic mutation			0.0006 **
Presence	19	57.9	
Absence	43	92.4	

** Statistical significance was set to *p* < 0.05.

**Table 4:** Multivariate Cox proportional hazards regression analysis for factors associated with postoperative recurrence of localized RCC.

Variables	Lower 0.95	Upper 0.95	Likelihood Ratio of Chi-Square	*p* Value
Tumor diameter	−1.425707	0.1053829	2.90251154	0.0884
Pathological T stage	−1.420865	0.2705481	1.90465633	0.1676
Fuhrman grade	−0.704015	1.1331817	0.16594524	0.6837
Somatic mutation in *ND*	0.3042389	1.8077773	8.11445592	0.0044 **

** Statistical significance was set to *p* < 0.05.

**Table 5:** Univariate analysis of factors predicting CSS after surgery.

Variables	No. of Cases	5-Year CSS Rate (%)	*p* Value
Age (years)			0.2085
≤60	28	100	
>60	34	94.1	
Sex			0.4682
Female	14	100	
Male	48	95.8	
Tumor localization			0.2481
Left	24	100	
Right	38	94.5	
Nephrectomy			0.3356
Total	42	95.2	
Partial	20	100	
Tumor diameter (mm)			0.0415 **
≤40	42	100	
>40	20	90.0	
Histological type			0.6373
clear	56	96.3	
non-clear	6	100	
pT stage			0.0330 **
≤pT2	57	98.2	
≥pT3	5	80.0	
Fuhrman grade			0.1272
I/II	54	98.1	
III/IV	8	87.5	
Vessel invasion			0.2890
Presence	50	91.7	
Absence	12	98.0	
*ND1* somatic mutation			0.0341 **
Presence	19	89.5	
Absence	43	100	

** Statistical significance was set to *p* < 0.05.

**Table 6:** Multivariate Cox proportional hazards regression analysis for factors associated with CSS after surgery of localized RCC.

Variables	Lower 0.95	Upper 0.95	Likelihood Ratio of Chi-Square	*p* Value
Tumor diameter	−43,798.79	43,776.674	2.20091038	0.1379
Pathological T stage	−2.119379	1.1119271	0.48646922	0.4855
Somatic mutation in *ND1*	−24,003.11	24,024.239	3.0118406	0.0827

**Table 7:** Multivariate Cox proportional hazards regression analysis for factors associated with RFS after surgery of localized ccRCC.

Variables	Lower 0.95	Upper 0.95	Likelihood Ratio of Chi-Square	*p* Value
Tumor diameter	−1.474545	0.0066134	3.41616475	0.0646
Fuhrman grade	−0.87471	0.7815922	0.01212236	0.9123
Somatic mutation in *ND1*	0.0945703	1.5221494	5.33970621	0.0208 **

** Statistical significance was set to *p* < 0.05.

## Supplementary Materials

Supplementary materials can be found at www.mdpi.com/1422-0067/17/12/2049/s1.
